# Contributing factors in judgment of fairness by monetary value

**DOI:** 10.1186/1471-2202-12-S1-P329

**Published:** 2011-07-18

**Authors:** David Nicoladie Tam

**Affiliations:** 1 Department of Biological Sciences, University of North Texas, Denton, TX 76203, USA

## 

Given that we have developed for emotional response (Emotional-Gain Model) [1-4] and a model for fairness (Fairness-Equity Model) [5] that quantified emotional bias and fairness bias, we will use these models to reveal the hidden factors contributing to the emotional bias and fairness bias. Using the Ultimatum Game (UG) with human subjects to split a sum of money, we compare the responses between sharing $10 vs. $10 million. The results show that the proportionality relationship in fairness perception is skewed according to the amount of money as well as the relative ratio of equity. The proportionality relationship in emotional response is also skewed by the amount of money and the relative ratio of disparity. This quantifies the specific fairness bias and emotional bias based on the monetary value of the disparity (or equity) between the shares. The biases are characterized by the shifting of the fairness-curve in the fairness-equity space, and the shifting of the emotional-curve in the emotional-gain space graphically. The result also reveals the objectivity of perception in spite of the subjectivity of their perception to inequitable share.
